# Skin chromophore mapping by smartphone RGB camera under spectral band and spectral line illumination

**DOI:** 10.1117/1.JBO.27.2.026004

**Published:** 2022-02-21

**Authors:** Ilona Kuzmina, Ilze Oshina, Laura Dambite, Vanesa Lukinsone, Anna Maslobojeva, Anna Berzina, Janis Spigulis

**Affiliations:** a University of Latvia, Institute of Atomic Physics and Spectroscopy, Biophotonics Laboratory, Riga, Latvia; b The Clinic of Laser Plastics, Riga, Latvia

**Keywords:** multispectral imaging, light-emitting diodes, lasers, image processing, tissues, biophotonics

## Abstract

**Significance:**

Multispectral imaging enables mapping of chromophore content changes in skin neoplasms, which helps to diagnose a pathology. Different types of light sources can be used for the imaging. Design of laser-based illuminators is more complicated and, consequently, they are more expensive than LED-based illuminators. On the other hand, spectral line illumination has the advantage of less complicated calculations, since only the discrete maximum wavelengths need to be considered. Spectral band and spectral line approaches for multispectral skin diagnostics have not been compared so far. This can help to evaluate the accuracy and effectiveness of both approaches.

**Aim:**

To compare two specific illumination modalities—spectral band and spectral line illumination—from the point of performance for mapping of *in vivo* skin chromophores.

**Approach:**

Three spectral images of the same skin malformations were captured by a smartphone RGB camera with two different add-on illuminators comprising LED emitters and laser emitters, respectively. Five types of benign skin neoplasms were included in our study. Concentrations of skin melanin, oxy- and deoxy-hemoglobin at image pixel groups were calculated using the Beer–Lambert law.

**Results:**

Skin chromophore maps and statistical analysis of mean concentrations’ changes in the neoplasms compared to the surrounding skin are presented and discussed. The data of the laser emitters led to significantly higher (∼10 times) increase of the oxy-hemoglobin values in vascular neoplasms and much lower deoxy-hemoglobin values, if compared to the data obtained by the LED emitters.

**Conclusions:**

Analysis of the obtained chromophore distribution maps and concentration variations in neoplasms led to conclusion that the spectral line illumination approach is more appropriate for this application. Considering only the peak wavelengths of illumination spectral bands leads to essentially different results if compared to those obtained by spectral line illumination and may cause misinterpretations in the clinical assessment of skin neoplasms.

## 1 Introduction

Early *in vivo* assessment of skin malformations is important for timely recognition and treatment of malignant neoplasms.^1^ The accuracy of diagnostics mainly depends on the experience of dermatologist while the “gold” standard for diagnostics remains histology of biopsied tissue samples. The content and depth of pigmentation is important, as melanocytes in deeper skin layers may indicate to malignancy of the neoplasm. Benign pigmented skin neoplasms are classified according to the location of melanocytes in the skin layers. For example, junctional nevi have melanocytes only in epidermis, combined nevi—both in the epidermis and dermis, dermal nevi—mainly in the dermis.^2^

A number of optical methods have been developed for noninvasive *in vivo* skin diagnostics, including confocal microcopy, fluorescence imaging, and optical coherence tomography.^3^ Recently, skin imaging by smartphones under white broadband illumination exploiting the embedded LEDs or ambient light was proposed as a diagnostic modality.^4^^–^^8^ There are smartphone apps that use the ABCDE criteria,^9^ commonly exploited by dermatologists, for analysis of the taken color images of skin neoplasms. However, only the color and shape features of the malformation cannot assure proper skin cancer diagnostics; the smartphone RGB image apps such as Google’s “dermatology assist tool” are heavily criticized by doctors.^10^

In addition, multispectral imaging studies that offered diagnostic solutions at spectrally specific illumination using narrowband optical filters or LEDs (at typical spectral bandwidth 10 to 40 nm) have been carried out.^7^^,^^11^^–^^13^ In most cases, only the peak wavelengths of spectral bands were taken into consideration during the image processing, so contribution of other wavelengths involved in the spectral band remained ignored. To the best of author’s knowledge, there are no published data that evaluate accuracy of such approach in comparison with “pure” single-wavelength approach. To fill the gap, this study was aimed at comparing results on chromophore mapping and chromophore concentration evaluation in various skin malformations, obtained at two illumination modalities—spectral band and spectral line illumination. Selected LEDs provided spectral band illumination while the spectral line illumination was performed by lasers supplemented with a special diffuser that ensured uniform illumination of the target area.^13^^,^^14^ Images of the skin malformations were captured by RGB camera of the same smartphone model.

The spectral images of examined skin malformations were processed using the Beer–Lambert law.^13^^,^^15^^–^^21^ An important parameter here is a mean path length (MPL) of skin backscattered photons. Previous studies^20^^,^^21^ used in calculations a mean light penetration depth in Caucasian skin^22^ or the MPL values obtained by Monte Carlo (MC) simulations.^13^ In this study, concentration increase or decrease of skin melanin, oxy-hemoglobin, and deoxy-hemoglobin in the malformations was calculated using experimentally estimated MPL values, and five types of benign skin neoplasms were examined.

## 2 Materials and Methods

### 2.1 In Vivo Measurements on Volunteers

*In vivo* measurements were performed on 79 volunteers with skin phototypes I, II, or III (Fitzpatrick classification), aged between 12 and 88; their written consent was obtained before the measurements. Each neoplasm was evaluated and classified by a certified dermatologist involved in this study (A. Berzina, one of the co-authors). The classification of the neoplasms and all measurements were performed in the House of Science, University of Latvia, under permission of a local Ethics Committee. Table 1 provides detailed description of the skin neoplasms included in this study. Pigmented (junctional, combined, and dermal nevi) and vascular (hemangiomas) neoplasms as well as seborrheic keratoses from different parts of the body were measured and analyzed. Most of them were located on the back and abdomen; some on the chest, neck, arms, and legs.

**Table 1:** Types and number of skin neoplasms.

**Diagnosis**	**Number**
Junctional nevus	19
Combined nevus	19
Dermal nevus	23
Seborrheic keratosis	23
Hemangioma	21
**Total**	**105**

**Fig. 1:**
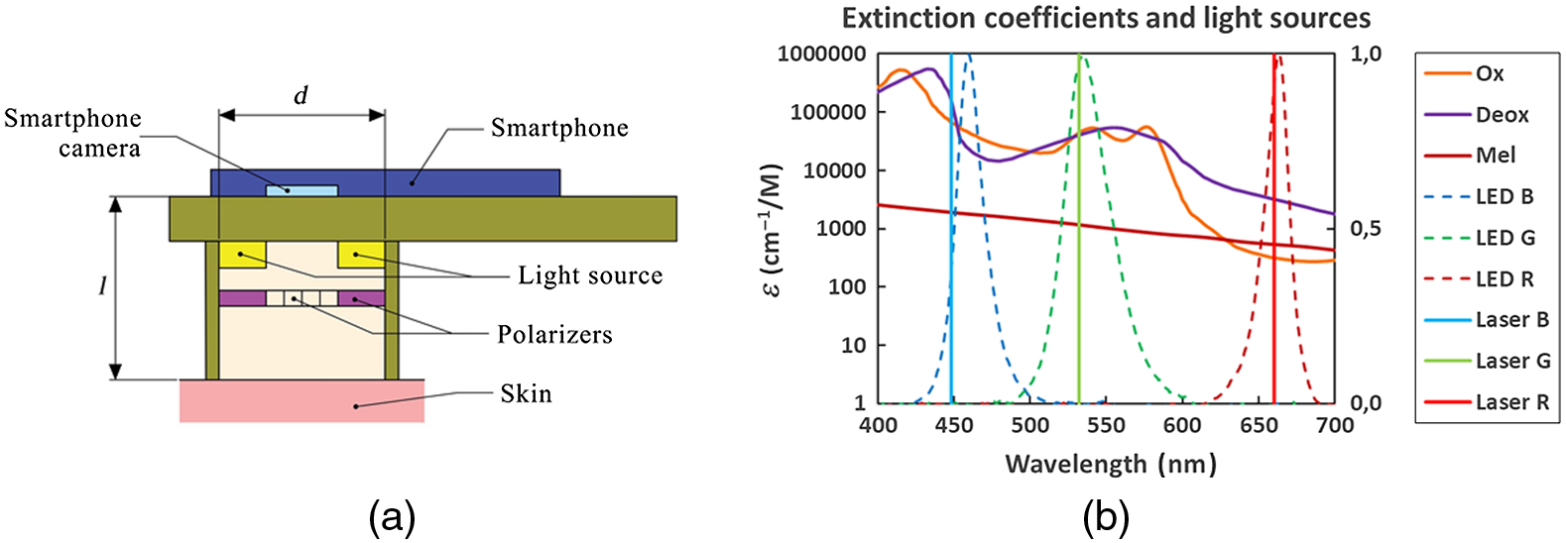
(a) Set-up scheme for multispectral imaging of skin diffuse reflectance by a smartphone camera. (b) Spectral dependence of extinction coefficients of three main skin chromophores (melanin, oxy- and deoxy-hemoglobin) and normalized spectra of the LED and laser illuminators. Ox, oxy-hemoglobin; Deox, deoxy-hemoglobin; Mel, melanin; LED B, blue LEDs; LED G, green LEDs; LED R, red LEDs; Laser B, blue laser; Laser G, green laser; Laser R, red laser.

**Fig. 2:**
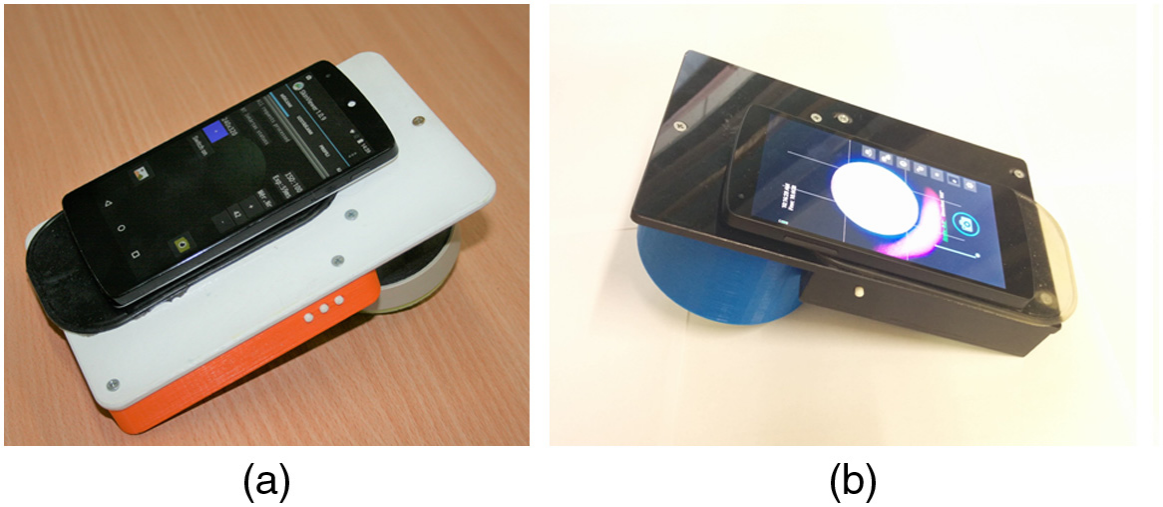
Appearance of (a) LED and (b) Laser prototypes.

A set-up used for skin diffuse reflectance image measurements is shown in Fig. 1(a). Diffuse reflectance RGB images were captured by a Google Nexus5 smartphone comprising 8 Mpx image sensor SONY IMX179 with known RGB-sensitivities.^23^ Two different add-on illuminators were used—one comprising a ring of three color LEDs (LED prototype)^12^ and the other comprising three wavelength lasers and a ring-shaped flat milky-Plexiglas diffuser (Laser prototype). Detailed design description of the Laser prototype is available in Ref. 13. The LED prototype provided sequential three-color continuous illumination by three types of LEDs [four diodes of each type, models: XBDROY-00-0000-000000L01 (peak value of emitted band at 460 nm, 500 mW, FWHM∼20  nm), CreeLED, Inc., ELSW-J11G3-0LPNM-DG1G3 (535 nm, 100 mW, FWHM∼35  nm), Everlight Electronics Co Ltd., LH CP7P-2T3T-1-0-350-R18 (663 nm, 355 mW, FWHM∼20  nm), OSRAM Opto Semiconductors Inc.] Each set of LEDs was powered separately by the LED driver, to provide similar illumination intensity and constant signal output. The Laser prototype ensured simultaneous three laser line (448, 532, and 659 nm) continuous illumination by three pairs of compact 20-mW power laser modules (models PGL-DF-450 nm-20 mW-15011564, PGL-VI-1-532 nm-20 mW-15030443, and PGL-DF-655 nm-20 mW-150302232, Changchun New Industries Optoelectronics Tech. Co., Ltd.) for capturing of three spectral line images by a single snapshot^12^ [Fig. 1(b)]. Microcontroller (STM32F103) managed operation of the three-laser power modules via control of the laser power supply unit. The following acquisition parameters were adjusted using ColorChecker^®^ White Balance (XRite) as a reference for each illumination wavelength of the LED prototype: intensities on the measured area: $I_{663\;\mathrm{nm}}=59\;\mu W/{\mathrm{cm}}^{2}$, $I_{535\;\mathrm{nm}}=38\;\mu W/{\mathrm{cm}}^{2}$, $I_{460\;\mathrm{nm}}=21\;\mu W/{\mathrm{cm}}^{2}$, an exposure time: 0.02 s, ISO=100, a white balance: 6000 K, whereas for the Laser prototype: the intensity on the measured area under simultaneous illumination of all wavelengths $∼40\;\mu W/{\mathrm{cm}}^{2}$, the exposure time: 0.87 s, ISO=100, the white balance: 6000 K. Image capture time was <3  s for the LED prototype including the switching time of the LEDs (∼20  ms at each illumination). RAW images captured by Laser prototype and JPG images captured by LED prototype were further used for analysis in MATLAB. Two smartphone apps—SkinViewer (self-developed) and AZ Camera (commercial)—were used for the LED and Laser prototypes, respectively. Both apps allowed adjusting the settings for the measurements. A black paper marker was used on the skin next to each neoplasm during the measurements by LED prototype for further image stabilization. The diameter of the imaging area of both illuminators was 4.0 cm; the distance between the smartphone camera lens and the target surface was 7.0 cm for the LED prototype and 8.0 cm for the Laser prototype. Crossed polarizers in front of the camera and illumination rings, respectively, were used to minimize the detection of reflected light from the skin surface. All measurements were performed with gentle pressure of the device on the skin by an experienced operator. A soft silicone contact ring mounted on the tip of the shielding cylinder [not shown in Fig. 1(a)] reduced the contact pressure. The appearance of both prototypes is shown in Fig. 2. The dimensions of the LED and the Laser prototypes are 11×18×6.5  cm (cylinder diameter 7 cm, field of view diameter 4 cm) and 11×18.5×7.5  cm (cylinder diameter 9 cm, field of view diameter 4 cm), respectively.

### 2.2 Estimation of the Mean Path Lengths of Skin Backscattered Photons

The MPL of skin-remitted photons is defined as the mean value of the travel distances of all backscattered photons detected at certain separation from their input. MPL for each working wavelength (460, 535, and 663 nm for the LED prototype, and 448, 532, and 659 nm for the Laser prototype) were estimated by linear approximation of our previously measured MPL data within the spectral range 520 to 800 nm for the input–output separation 1 mm. This separation was assumed to correspond the measurement conditions of the skin diffuse reflectance images where a part of photons detected at any pixel have diffused from the neighboring regions of skin. Only the peak wavelengths of illumination spectral bands were considered in this study. The MPL measurement methodology was based on the skin-remitted photon time-of-flight determination in the picosecond range at selected spectral bands and source–detector fiber separations. Processing of the measured data involved comparing the shapes of skin input and output pulses—a(t) and b(t), respectively. The temporal distribution function f(t) of photon arrivals following infinitely narrow δ-pulse input was found by deconvolution of the integral:

$$b(t)=\int_{0}^{t}a(t-\tau )f(\tau )d\tau .$$ (1)

**Fig. 3:**
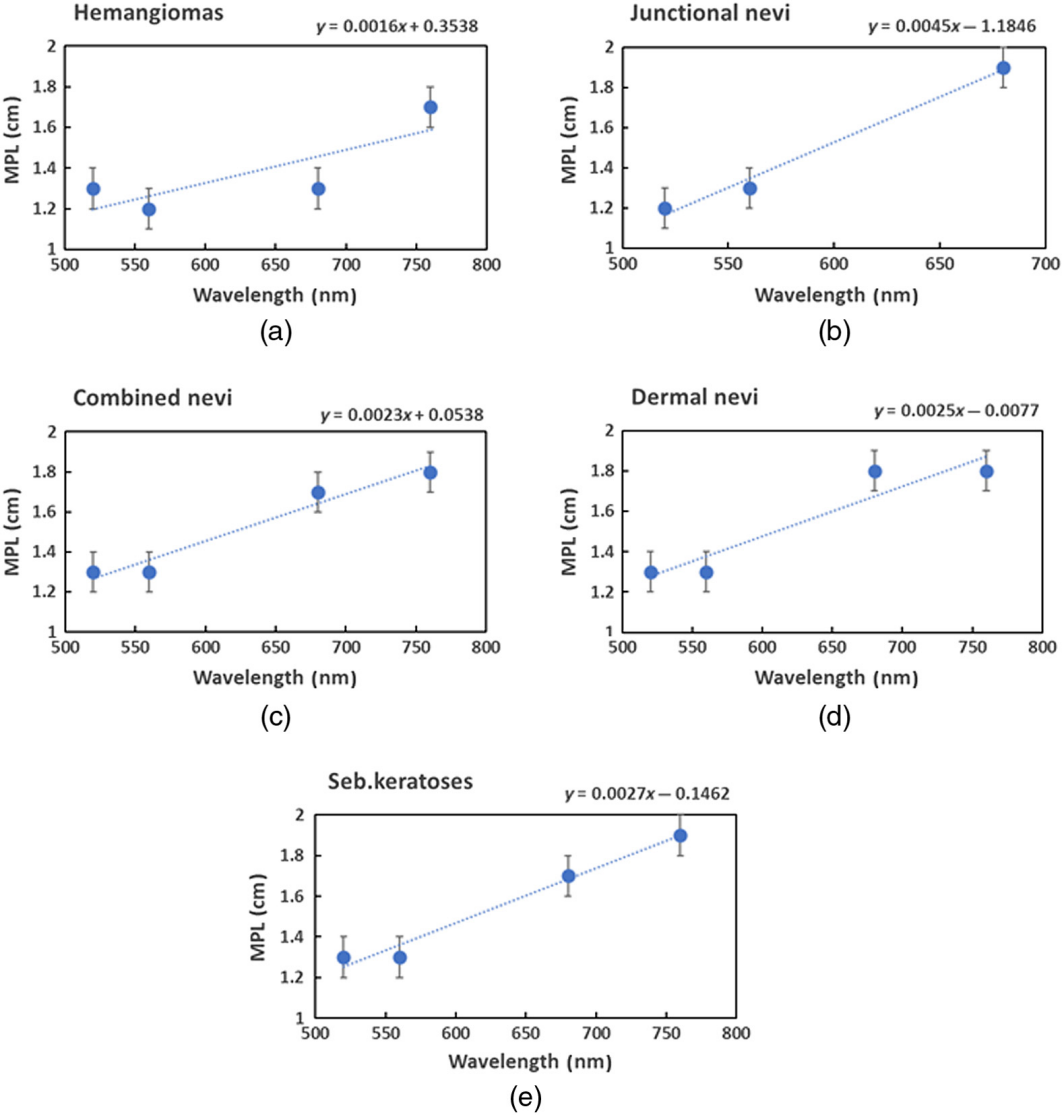
Experimentally estimated MPLs of the skin-remitted photons at the source–detector distance 1 mm for (a) hemangiomas, (b) junctional, (c) combined, and (d) dermal nevi, and (e) seborrheic keratoses.

Photon path length in skin was calculated as

$$\phi (s)=f(t)\cdot c_{v}/n,$$ (2)

where $c_{v}$ is the speed of light in vacuum and n is the mean refraction index of superficial skin tissues (n∼1.4).^24^^,^^25^ The mean values of integrated path length distribution functions were considered as the photon MPLs. Detailed description of the experimental set-up and the MPL measurement methodology is presented in Ref. 26. The initial data taken from healthy forearm skin^26^ were complemented by the measured MPL from various skin malformations (Fig. 3). The approximation was performed using built-in function of MS Excel for the mean experimental values related to each type of neoplasms.

**Fig. 4:**
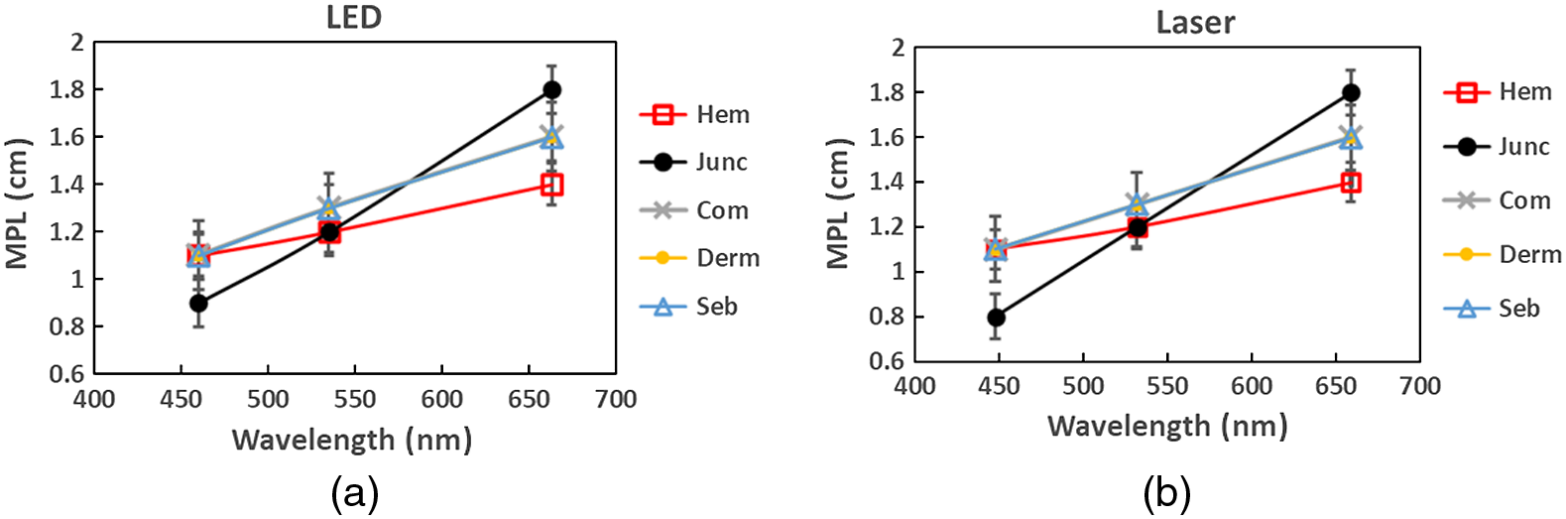
Estimated spectral dependences of MPL for junctional (Junc), combined (Com) and dermal (Derm) nevi, seborrheic keratoses (Seb), and hemangiomas (Hem) at the peak wavelengths of the (a) LED and (b) Laser prototypes.

Figure 4 shows the estimated spectral dependences of the specific MPLs for junctional (Junc), combined (Com), and dermal (Derm) nevi, seborrheic keratoses (Seb), and hemangiomas (Hem) obtained from the linear approximation. The results are similar for both prototypes. The mean photon pathlengths increase at longer wavelengths for all types of neoplasms. The MPL values for seborrheic keratoses combined and dermal nevi coincide at each of the peak wavelengths. The MPL values of junctional nevi are higher at red wavelengths and lower at blue wavelengths compared to other neoplasms, whereas the MPL values of hemangiomas are lower at red wavelengths and coincide with the values of seborrheic keratoses, combined and dermal nevi at blue wavelengths.

Since we exploited only the peak wavelengths of the LED spectral bands in the calculations, the MPL differences between the peak wavelength and those at the band wings where intensity dropped down to 10% of its maximum were also analyzed to evaluate possible errors due to this simplification. The 10% values correspond to the wavelength pairs 440 and 488 nm, 505 and 579 nm, and 638 and 680 nm. The estimated errors due to the differences of MPL were found to be in the range 3% to 14% for blue illumination, 4% to 16% for green illumination, and 2% to 6% for red illumination, depending on the type of neoplasms. The highest MPL differences were found for the junctional nevi: 10% at 440 nm and 14% at 488 nm, 11% at 505 nm and 16% at 579 nm, and 6% at 638 nm and 4% at 680 nm. The lowest MPL differences were related to hemangiomas: 3% and 4% for blue illumination, 4% and 5% for green illumination, and 3% and 2% for red illumination.

### 2.3 Image Processing

**Fig. 5:**
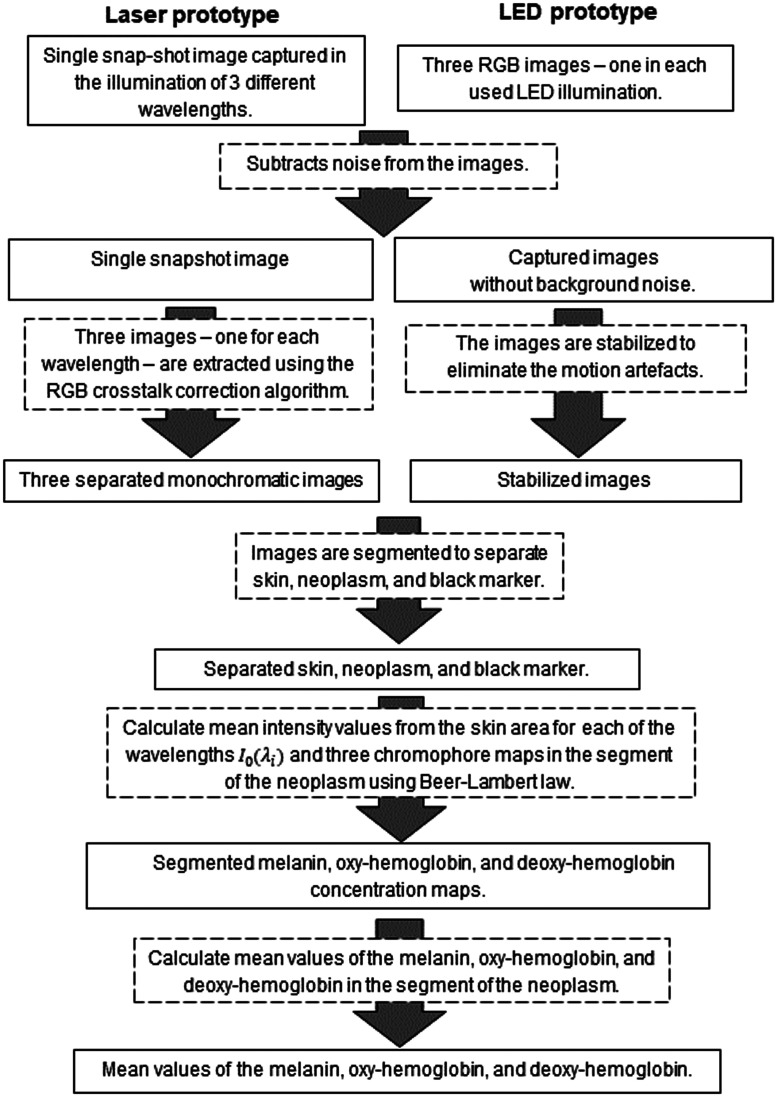
Image processing flowchart.

Image processing was performed in MATLAB as shown in Fig. 5. A noise was removed from images by the dark image subtraction. Three images—one for each used wavelength—were extracted from skin diffuse reflectance image of the Laser prototype using RGB crosstalk correction algorithm.^14^ Stabilization of the three LED images was performed using built-in functions normxcorr2 (normalized 2D cross correlation) and imtranslate (translate image). The “green” image and black marker that was placed on the skin near the neoplasm during the measurements were used for stabilization. The segmentation of the neoplasm, surrounding skin, and marker (in the case of the LED prototype) were performed using the built-in k-means clustering function. Mean value of the reflected intensity from the surrounding healthy skin segment $I_{0}$ was used as a reference.

Chromophore maps were calculated using a modified Beer–Lambert law for each working wavelength:

$$I=I_{0}\;\exp(-\mu_{a}l),$$ (3)

where $\mu_{a}$ is an absorption coefficient, I and $I_{0}$ are the intensities of diffusely reflected light from the neoplasm and surrounding skin, respectively, and l is the MPL of skin-remitted photons.

An algorithm used in a previous investigation^13^ was applied for the three exploited wavelengths with respect to the three considered skin chromophores (melanin, oxy-hemoglobin, and deoxy-hemoglobin):

$$\left\{ \begin{array}{c} c_{\mathrm{Mel}}\varepsilon_{\mathrm{Mel}}(\lambda_{1})+c_{\mathrm{Ox}}\varepsilon_{\mathrm{Ox}}(\lambda_{1})+c_{\mathrm{Deox}}\varepsilon_{\mathrm{Deox}}(\lambda_{1})=\frac{\ln\;\frac{I_{0}(\lambda_{1})}{I(\lambda_{1})}}{2.303l(\lambda_{1})}\\ c_{\mathrm{Mel}}\varepsilon_{\mathrm{Mel}}(\lambda_{2})+c_{\mathrm{Ox}}\varepsilon_{\mathrm{Ox}}(\lambda_{2})+c_{\mathrm{Deox}}\varepsilon_{\mathrm{Deox}}(\lambda_{2})=\frac{\ln\;\frac{I_{0}(\lambda_{2})}{I(\lambda_{2})}}{2.303l(\lambda_{2})}\\ c_{\mathrm{Mel}}\varepsilon_{\mathrm{Mel}}(\lambda_{3})+c_{\mathrm{Ox}}\varepsilon_{\mathrm{Ox}}(\lambda_{3})+c_{\mathrm{Deox}}\varepsilon_{\mathrm{Deox}}(\lambda_{3})=\frac{\ln\;\frac{I_{0}(\lambda_{3})}{I(\lambda_{3})}}{2.303l(\lambda_{3})} \end{array},\right.$$ (4)

where Mel is the melanin, Ox is the oxy-hemoglobin, Deox is the deoxy-hemoglobin, ε is the extinction coefficient, and c is the changed concentration of the chromophore in the neoplasm in comparison with the surrounding skin. The coefficient 2.303 represents the ln(10) and is introduced due to conversion of the molar extinction coefficient to the absorption coefficient. In the case of the LED prototype, only the peak wavelengths’ values of LED-emitted spectral bands were considered.

The mean values of the changed chromophore concentrations were calculated from the segmented chromophore maps; the surrounding healthy skin was used as the reference ($I_{0}$) at all considered wavelengths to exclude the errors due to the variability of the tissue scattering levels among the samples.

## 3 Results

**Fig. 6:**
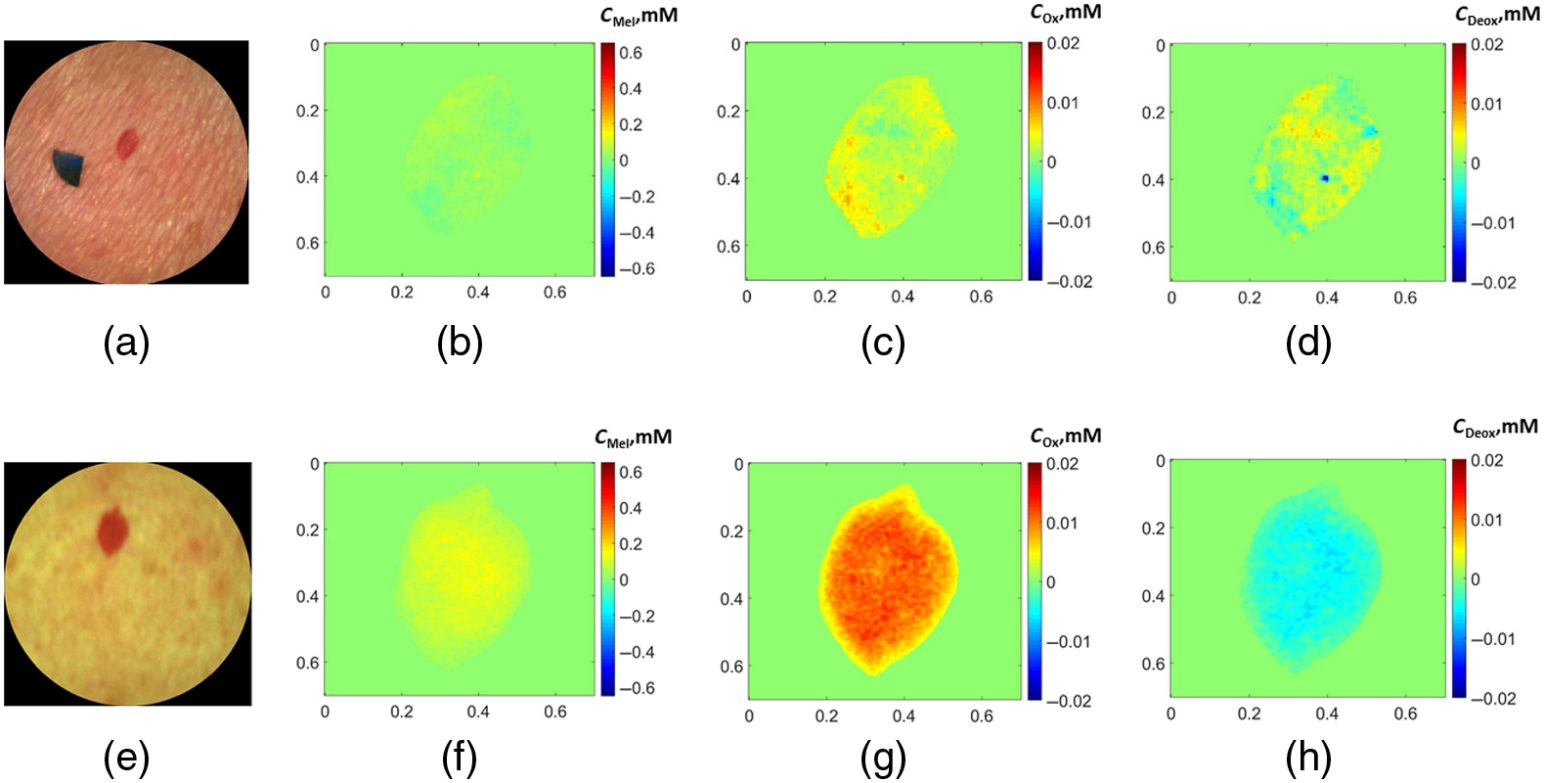
Example of RGB images of (a), (e) a hemangioma and the corresponding segmented chromophore maps of (b), (f) melanin, (c), (g) oxy-hemoglobin, and (d), (h) deoxy-hemoglobin for the (a)–(d) LED prototype and (e)–(h) Laser prototype. x and y axes indicate the size of the map in centimeters; color bar, the concentration changes of the chromophores in millimoles; $c_{\mathrm{Mel}}$, melanin concentration; $c_{\mathrm{Ox}}$, oxy-hemoglobin concentration; and $c_{\mathrm{Deox}}$, deoxy-hemoglobin concentration.

**Fig. 7:**
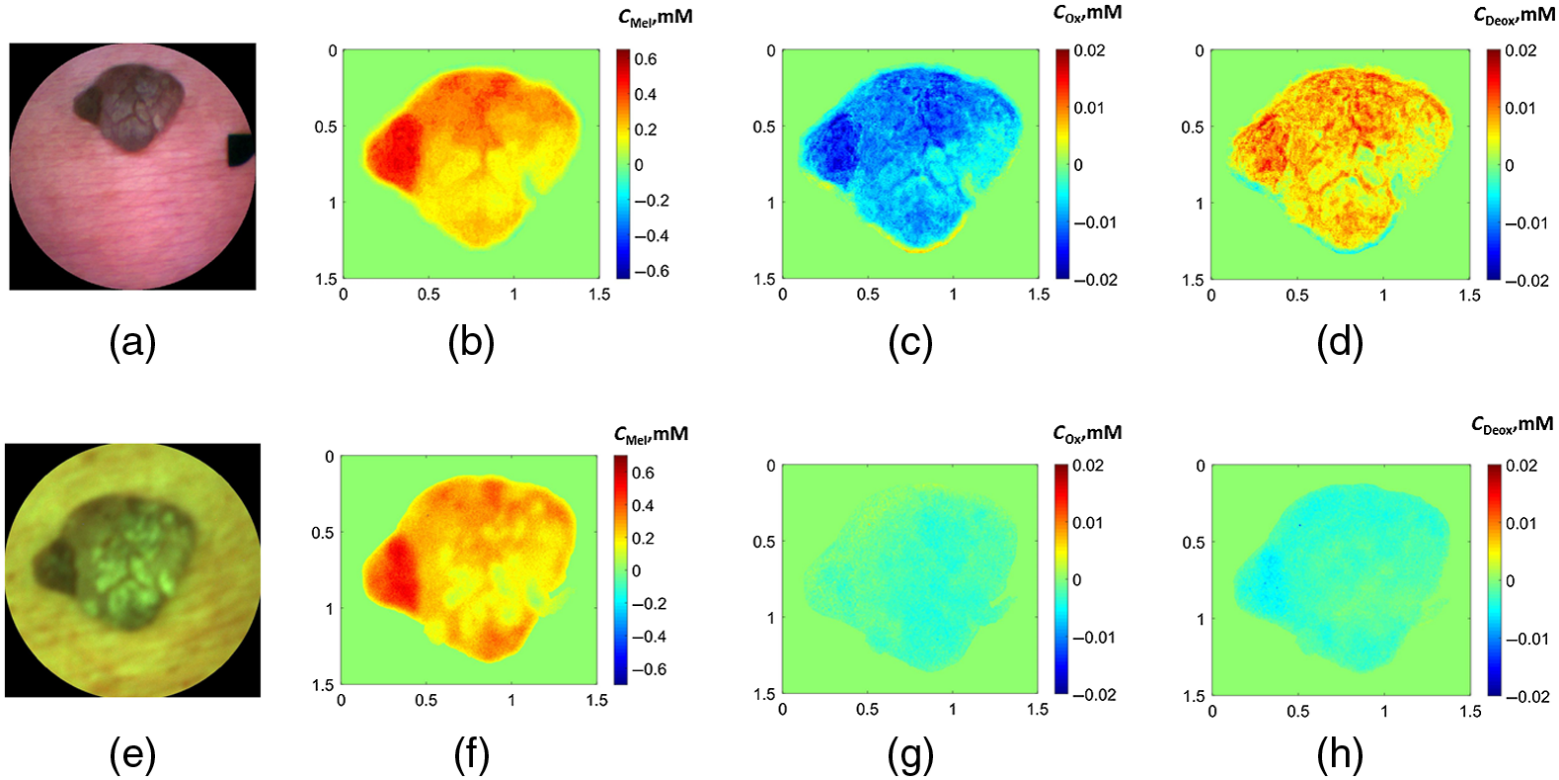
Example of RGB images of (a), (e) a seborrheic keratosis and the corresponding segmented chromophore maps of (b), (f) the melanin, (c), (g) oxy-hemoglobin, and (d), (h) deoxy-hemoglobin for the (a)–(d) LED and (e)–(h) Laser prototypes. x and y axes indicate the size of the map in centimeters; color bar, the concentration changes of the chromophores in millimoles; $c_{\mathrm{Mel}}$, melanin concentration; $c_{\mathrm{Ox}}$, oxy-hemoglobin concentration; and $c_{\mathrm{Deox}}$, deoxy-hemoglobin concentration.

Figures 6 and 7 illustrate examples of RGB images obtained by both devices and the corresponding segmented chromophore maps of a hemangioma and a seborrheic keratosis. The segmented maps illustrate the region of the neoplasm from which the mean values of the chromophores were calculated while the surrounding skin values are set to zero. The maps show higher concentration of the melanin compared to the surrounding skin for both prototypes [Figs. 6(b) and 6(f)]. In the case of the Laser prototype [Figs. 6(g) and 6(h)], a more pronounced increase of oxy-hemoglobin and decrease of deoxy-hemoglobin concentrations is observed in hemangiomas if compared to the LED prototype [Figs. 6(c) and 6(d)].

The example in Fig. 7 shows the increased concentration of melanin in seborrheic keratosis (compared to the surrounding skin) for both prototypes. The concentrations of both oxy- and deoxy-hemoglobin are slightly reduced in the images of Laser prototype [Figs. 7(g) and 7(h)], whereas the increased concentration of deoxy-hemoglobin and the decreased oxy-hemoglobin concentration is observed in the images taken by the LED prototype [Figs. 7(c) and 7(d)]. One should note that most of the seborrheic keratoses have inhomogeneous distribution of the chromophore concentration over the area and may have both positive and negative values of chromophore concentrations’ changes relatively to the healthy skin. Other types of neoplasms showed more homogeneous distribution with distinct positive or negative values.

**Fig. 8:**
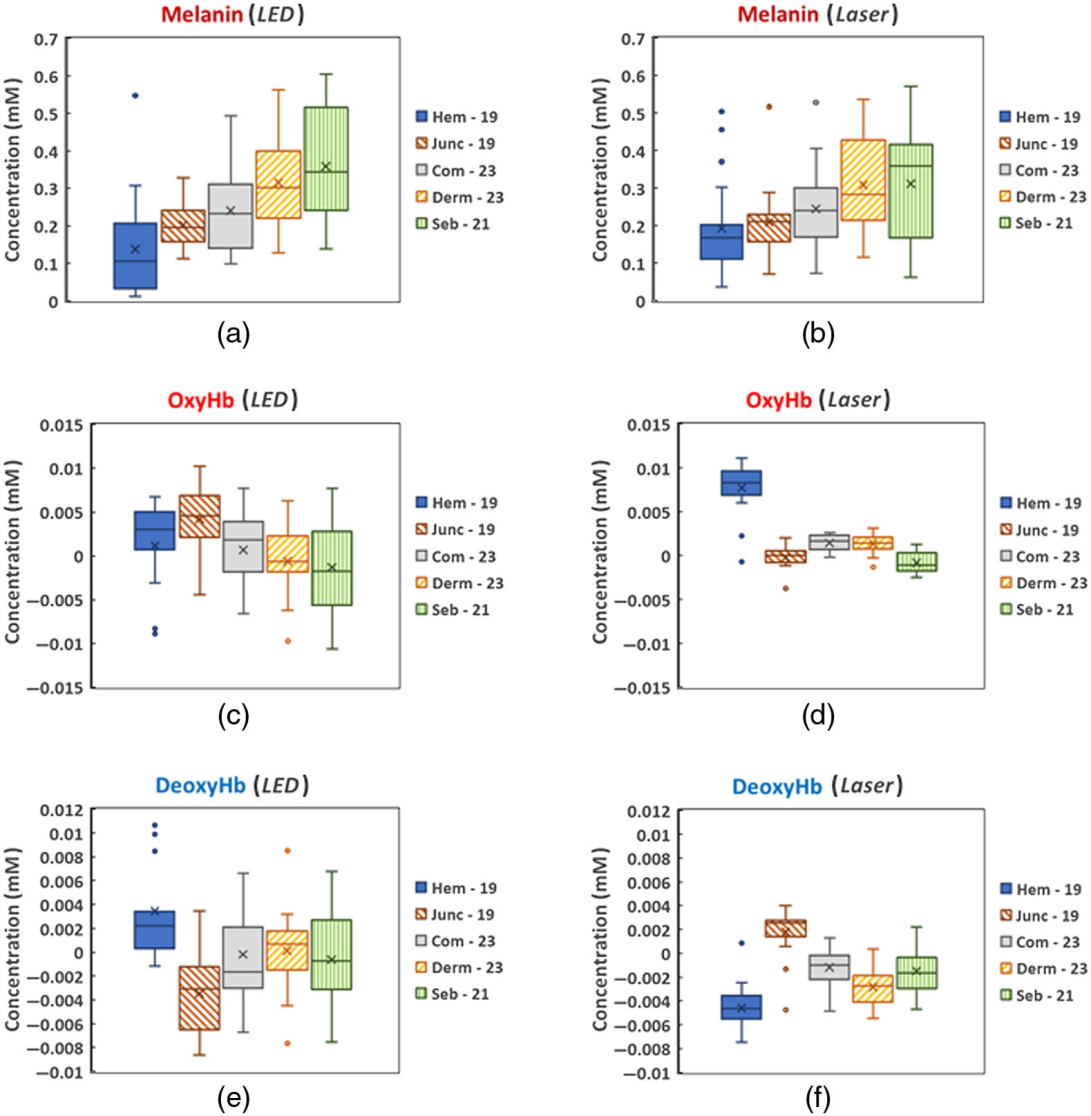
Comparison of the melanin, oxy- and deoxy-hemoglobin concentrations’ changes calculated from the images of (a), (c), (e) the LED and (b), (d), (f) Laser prototypes: Hem, hemangiomas; Junc, junctional nevi; Com, combined nevi; Derm, dermal nevi; and Seb, seborrheic keratoses. The edges of the boxes denote 25th to 75th percentile value; solid line inside the box, median value; cross, mean value; bars above and below the box, standard deviation; and points represented by circles, outliers.

Figure 8 presents a comparison of the chromophore concentrations’ increase/decrease in the neoplasms calculated from the images of the (a), (c), (e) LED and (b), (d), (f) Laser prototypes, using MPL values for each type of neoplasms as presented in Fig. 4. Negative oxy- or deoxy-hemoglobin concentrations indicate that they have decreased if compared to the surrounding healthy skin.

### 3.1 Melanin

All types of the neoplasms show a positive (increased) concentration of melanin in the range from 0.1 to 0.5 millimoles (mM) at both illuminations [Figs. 8(a) and 8(b)]. All hemangiomas exhibit the lowest increase in the melanin concentration and are well separated from the dermal nevi (25th to 75th percentile boxes do not overlap). The dermal nevi and seborrheic keratoses show the highest increase in the melanin concentration, whereas the data for seborrheic keratoses overlap with those for all types of the pigmented nevi.

### 3.2 Oxy-Hemoglobin

Figure 8(d) shows that the hemangiomas, on the other hand, exhibit the highest positive values of the oxy-hemoglobin concentration and are significantly separated from other types of the neoplasms if images of the Laser prototype are processed; the values of the increase are in the range from 0.007 to 0.01 mM. Most of the seborrheic keratoses have negative (decreased) oxy-hemoglobin values and they are well separated from all other groups. The junctional nevi show lower changes of the oxy-hemoglobin concentration compared to the dermal and combined nevi.

In the case of the LED prototype [Fig. 8(c)], the value ranges overlap for all types of neoplasms. Most of the hemangiomas and junctional nevi have positive values while the combined and dermal nevi, and the seborrheic keratoses—both positive and negative.

### 3.3 Deoxy-Hemoglobin

The statistical distribution of the deoxy-hemoglobin concentration shows significant separation of the junctional nevi from the other pigmented neoplasms with the highest positive values for the data of the Laser prototype [Fig. 8(f)]. The values are in the range from 0.001 to 0.003 mM. Most of all other types of the neoplasms have negative values (decreased concentrations) with the lowest ones for the hemangiomas which are also well separated from the combined nevi and seborrheic keratoses.

As for the data of the LED prototype [Fig. 8(e)], the hemangiomas show positive values and are well separated from the junctional nevi which have the lowest negative values. The other types of neoplasms have both positive and negative values, and the value ranges overlap with the ranges of the hemangiomas and junctional nevi.

## 4 Discussion

Overall, results obtained using the Laser prototype (spectral line illuminator) showed better discrimination between the vascular and pigmented neoplasms if compared to the LED prototype (spectral band illuminator) in terms of the oxy- and deoxy-hemoglobin concentration changes (see Fig. 8). Also concerning pure vascular neoplasms–hemangiomas, Laser prototype data led to significantly higher (∼10  times) increase of the oxy-hemoglobin values and much lower deoxy-hemoglobin values, if compared to the data obtained by the LED prototype (Figs. 6 and 8). Our previous study of agar-based skin optical phantoms^27^ showed that accuracy of the hemoglobin concentration estimation is 90% to 99% for the Laser prototype and 70% to 80% for the LED prototype. A t-test of the oxy- and deoxy-hemoglobin mean values showed statistically significant differences (p<0.05) between the pairs of the most types of neoplasms for Laser prototype. The exceptions are the pairs of combined–dermal nevi and junctional nevi–seborrheic keratoses if the oxy-hemoglobin concentrations are compared, and the pairs of the combined nevi–seborrheic keratoses and dermal nevi–seborrheic keratoses if deoxyhemoglobin concentrations are compared. Similar statistically significant differences (p<0.05) were observed for junctional nevi compared to other types for all three chromophores (except the pairs of junctional nevi–hemangiomas and junctional–combined nevi) and hemangiomas compared to other types for melanin and deoxy-hemoglobin concentrations obtained from the LED. Since only peak wavelengths of the LED-emitted spectral bands were considered, leaving the spectral band shapes without notice, the estimated concentrations may be less accurate compared to the data of Laser prototype. One can conclude that the other spectral components of LED-illuminated bands obviously also contribute and have to be taken into consideration when processing spectral band images, otherwise mistaken data on chromophore concentrations and their distributions can be obtained. It could be done, for instance, by approximating the spectral band with a certain number (for example, 10) of discrete spectral lines, each with specific relative intensity. If so, this number of Eq. (2) has to be solved for each spectral band, which makes the image processing procedure much more time-consuming and requires more computing resources. From this point, spectral line imaging approach by means of devices like the Laser prototype demonstrates essential advantages. On the other hand, design of laser-based illuminators is a bit more complicated and, consequently, they are more expensive than the LED-based illuminators. Our experience with laser-based prototypes,^13^^,^^14^^,^^21^^,^^28^ however, indicates that this kind of devices could be fully affordable for private clinics and general practitioners (total component price ∼ 1 k$).

As expected, the increase in melanin concentrations obtained in all pigmented neoplasms is higher than that of oxy-hemoglobin. However, calculations in frame of the Beer–Lambert model resulted in slight increase of the melanin concentrations also in several hemangiomas which are purely vascular malformations. Most probably, there are some specific limitations of this model which were not considered and must be studied in more details in the future.

One should note that exact values of MPL used in spectral image processing did not impact the main goal of this study—to compare performances of spectral band and spectral line illumination for chromophore assessment in skin malformations. We used the experimentally determined MPL values for spectral image processing, assuming them to be the most reliable data by now—but no way being universal for all clinical cases. For instance, the location of the neoplasm on the body could have an impact on the photon path lengths depending on the thickness of the fat layer and soft tissues beneath the skin; this is a matter for a separate study. All malformations of the same type were processed using the same MPL value which could differ from case to case depending on individual features. Despite that, the results showed relatively good discrimination between different neoplasms in terms of concentration variations of the main skin chromophores, especially when the Laser prototype was used (Fig. 8). One could wonder about relatively high values of the measured MPL (∼1 to 2 cm) if compared with those simulated in the frame of MC or other models.^13^ It is important to understand that here we presented the measured MPL which related only to the remitted (backscattered) photons, not to all skin-launched photons. Clearly, the remission related MPL values should be higher than those related to all (including absorbed) photons travelling in the skin tissues. In addition, the MC models may use larger numerical aperture values of the receiving fiber than in a real experiment where the fiber tip can be in good contact with the skin (n∼1.4). In such situation, mainly those photons travelling collinear to the fiber axis (i.e., orthogonal to the skin surface) are detected, and their path lengths certainly are longer than the statistically averaged path length of all launched photons. We verified that the temporal widening of the laser pulse in the detection fiber (which could lead to mistaken results) did not take place under experimental conditions.

Another direction for the future is the creation of a clinically accessible database with measured MPL values for skin-remitted photons related to different locations of the body and to patients of different complexions. Such a database can be created mainly by researchers and engineers considering the specific requirements for the MPL measurement equipment (broadband picosecond laser, TCSPC system, and fiber-optic contact probes^26^), which is available only in research laboratories.

## 5 Summary

Results of this paper demonstrate the advantages of spectral line imaging technology over the spectral band imaging if applied for the skin chromophore mapping and chromophore concentration evaluation in skin neoplasms. The data obtained by means of the laser illuminator demonstrated much better separation between the pigmented and vascular types of neoplasms, if compared to those obtained by the LED illuminator. Consequently, the approach previously used to process the spectral band images, considering only the maximum wavelength of the band, has proved to be inappropriate—other spectral components of the bands should be respected, as well. Errors due to the differences in MPL related to the wavelengths at the illumination band centers and wings (at 10% level) were estimated to be in the range 3% to 14% for blue illumination, 4% to 16% for green illumination, and 2% to 6% for red illumination, depending on the type of neoplasms. Although a relatively old smartphone model was used in this study, we believe that the results presented are applicable also to the latest smartphone models using apps with the possibility to manually adjust most of the settings, for example, ISO, exposure time, and white balance. To avoid misdiagnoses, spectral line imaging is recommended for the clinical evaluation of skin malformations. Since the surrounding skin is used as a reference and there is no single approach for determining the MPLs, the proposed method is more suitable for classification of skin neoplasms than for the absolute concentrations’ measurements. Additional studies should be performed on a larger size of groups including malignant neoplasms to establish the diagnostic criteria and to evaluate the accuracy of the method.

## Code, Data, and Materials Availability

The code and data underlying the results presented in this paper are not publicly available at this time but may be obtained from the authors upon reasonable request. No materials were used for the analysis.
